# Hyperglycaemia and oxidative stress upregulate HSP60 & HSP70 expression in HeLa cells

**DOI:** 10.1186/2193-1801-2-431

**Published:** 2013-09-03

**Authors:** Luke Hall, Ryan D Martinus

**Affiliations:** Department of Biological Sciences, Faculty of Science & Engineering, The University of Waikato, Private Bag 3105, Hamilton, New Zealand

**Keywords:** HSP60, HSP70, Mitochondria, Diabetes mellitus, Hyperglycaemia, Oxidative stress

## Abstract

Heat Shock Proteins 60 & 70 (HSP60 & HSP70) are intracellular protein that has been shown to be present at elevated levels in systemic circulation in Type 2 Diabetes mellitus (T2DM) patients. Conditions that lead to its secretion, and the mechanism of its translocation from cells, have not yet been defined. The aim of this study was to determine if specific cell stressors associated with T2DM, namely hyperglycaemia and oxidative stress, result in the upregulation of HSP60 in human cells in vitro. Human HeLa cells were grown in media supplemented with 100 mM glucose, 200 μM hydrogen peroxide (H_2_O_2_), and 50 μM sodium azide. Initially, the effect of these treatments on cell growth rate was examined, with each treatment significantly inhibiting growth rate. LDH and MTT assays were also used to successfully demonstrate that these treatments do not significantly increase cell lysis, but do significantly impair mitochondrial dehydrogenase activity. To confirm this mitochondria specific form of inhibition, DCFDA assay were used to investigate any increases in intracellular reactive oxygen species (ROS) generation. All three treatments resulted in significantly increased ROS generation, with greater ROS production occurring with a greater exposure time. Interestingly, when the protein levels of HSP60 and HSP70 were measured after 3 and 7 days of exposure of the HeLa cells to 100 mM glucose, 200 μM H_2_O_2_, and 50 μM sodium azide significant induction of these two molecular stress proteins were observed ranging from 2.43-5.08 fold compared to untreated control cells.

## Introduction

T2DM is the most common metabolic disease in the world, and the World Health Organisation (WHO) has predicted that T2DM related deaths will double between 2005 and 2030 (Danaei et al. 2011). Patients with T2DM have an increased risk of cardiovascular disease (CVD), which is responsible for 50-80% of deaths in people with diabetes (Danaei et al. 2011).

T2D has been found to be associated with mitochondrial specific cell stress (Giacco and Brownlee, 2010). The primary stressors that elicit this response in T2D are hyperglycaemia and oxidative stress (Mulder and Ling, 2009; Kim et al., 2008; Lowell and Shulman, 2005). T2D has also been found to be associated with elevated levels of heat shock protein 60 (HSP60) (Aguilar-Zavala et al., 2008; Nakhjavani et al., 2010; Yuan et al., 2011). HSPs are a class of ubiquitously expressed and func-tionally related proteins found in all living organisms. Their expression is increased in response to various cellular stressors in what is referred to as the heat shock response (Ritossa, 1996). The induction of one particular HSP, HSP60, has been found to be correlated with mitochondria specific cell stress (Martinus et al., 1996). Recently HSP60 has been found to be secreted and expressed extracellularly, after first being thought to be strictly intracellular where it performs roles as a chaperone and in protein folding (Yuan et al., 2011). However, these mechanisms of translocation and secretion have not been clearly identified. Extracellularly expressed HSP60 is believed to contribute to atherosclerotic development (Ellins et al., 2008; Pockley et al., 2003).

It is therefore plausible that the stressors associated with T2D are responsible for the elevated levels of extracellular HSP60 seen in T2D (Nakhjavani et al., 2010; Yuan et al., 2011). To determine if hyperglycaemia and oxidative stress results in the induction of HSP60 expression, HeLa cells were grown in media supplemented with glucose and hydrogen peroxide to simulate hyperglycaemia and oxidative stress respectively. Sodium azide was used as a third treatment as it is a known mitochondrial stressor, irreversibly binding to the heme component of complex IV in the electron transport chain. LDH and MTT assays were also implemented to show that the selected concentrations of glucose, hydrogen peroxide, and sodium azide did not lead to cell lysis, but did inhibit mitochondrial bioenergetics functions. ROS generation was also measured using a DCFDA assay, as evidence in the literature suggests that ROS activity mediates the mechanisms of HSP induction (Ahn and Thiele, 2002). HeLa cells were used as a representation of a peripheral tissue.

## Methods

### Cell culture and dose response

HeLa cells were grown in complete DMEM media at 37°C, 5% CO_2_, in a humidified incubator (standard incubation conditions). Cells were passaged every 7 days, and media was changed 3 days after passage. For the dose response experiments, cells were seeded at a density of approximately 60,000 cells/mL onto 24 well plates containing a range of glucose (25-125 mM), H_2_O_2_ (0-250 μM), and sodium azide (0-100 μM) concentrations. Cell density was estimated by the trypan blue exclusion method every 24 hours over a 7 day period.

### LDH assay

Near confluent HeLa cells were seeded onto a 96-well plate in 100 μL of DMEM supplemented with glucose (25-125 mM), H_2_O_2_ (0-250 μM), and sodium azide (0-100 μM). The plate was then returned to incubate in standard incubation conditions for 3 days, when the cells would be in the exponential growth phase. After which, one set of control cells (maximum control) was treated with 100 μL 10× lysis solution and the plate was returned to standard incubating conditions for 45 minutes. After this time period, the plate was centrifuged at 250×*g* for 5 minutes at room temperature (RT) to pellet cell debris. 50 μL of the supernatant from each well of the culture plate was transferred to the corresponding well of a 96-well enzymatic assay plate. 50 μL of reconstituted substrate was then added to the supernatant in each well of the assay plate. The plate was then covered from light and incubated at RT for 30 minutes. The assay was terminated by the addition of 50 μL of Stop Solution to each well of the plate and the absorbance was read at 490 nm.

### MTT assay

Near confluent HeLa cells were seeded onto a 96-well plate in 100 μL of DMEM supplemented with glucose (25-125 mM), H_2_O_2_ (0-250 μM), and sodium azide (0-100 μM). The plate was then returned to incubate in standard incubation conditions for 3 days, when the cells would be in the exponential growth phase. After 72 hours, the media was removed and replaced with 10 μL reconstituted MTT in DMEM. The plate was then incubated at standard incubation conditions for 2 hours. After the incubation period, the culture fluid was removed. This was followed by the addition of 100 μL MTT Solubilisation Solution. The plate was then placed on a minishaker for 10 minutes to assist in dissolving the crystals, and the absorbance was read at 570 nm and the background read at 655 nm.

### DCFDA assay

Near confluent HeLa cells were seeded onto a 96-well black culture plate in 100 μL of DMEM supplemented with glucose (25-125 mM), H_2_O_2_ (0-250 μM), and sodium azide (0-100 μM). The plate was then returned to incubate in standard incubation conditions for time periods of 24 hours, 3 days, and 7 days. At the end of the incubation period, the DMEM media was removed and the cells were washed with 100 μL pre-warmed PBS. The cells were then incubated for a further hour in 100 μL Opti-MEM containing only 2% FBS and further supplemented with 2.5 μL of 400 μM DCFDA. After the one hour incubation period, the media was again removed and the cells washed twice with 100 μL PBS. 200 μL Opti-MEM was then added, containing 2% FBS, and the fluorescence intensity over a 30 minute period at excitation and emission wavelengths of 485 nm and 520 nm respectively were determined. Data was standardised to the control analysis done in the absence of H_2_O_2_ (at the three different incubation times (24 h, 3 & 7 days)) and then expressed as relative fluorescence units to the controls.

### Heat shock control cells

As a means of obtaining a positive control a heat shock treatment was utilised (Martinus et al., 1996). HeLa cells were grown in a culture flask for 3 days, so as to reach the exponential growth stage. The media was then replaced with DMEM pre-warmed to 45°C, and the flask was placed into a 45°C incubator for 20 minutes. Following heat treatment, the media was again replaced, this time with DMEM pre-warmed to 37°C. The culture was returned to standard incubating conditions for 6 hours. After this time, the cells were harvested for protein extraction.

### Protein extraction

Total protein was isolated using TENT buffer and freshly added 0.4 mM phenylmethylsulfonyl fluoride (PMSF) as a protease inhibitor. Samples were ruptured with 100 μL TENT buffer by vortexing, and were subsequently incubated at 4°C for 30 minutes. After this incubation period, samples were spun at 10,000 rcf for 5 minutes at 4°C to pellet cell debris. The supernatant containing whole protein was transferred to a new tube and stored at −20°C.

### Protein separation and transfer

Total protein was separated on a 10% polyacrylamide discontinuous gel (0.76 mm) and run in a Mini-Protean 3 Cell gel tank.

Each sample containing 20 μg total protein was made up to 20 μL with protein loading buffer. The samples were denatured in boiling water for approximately 5 minutes before loading into the wells. The gel was electrophoresed at a constant 20 mA as the bands ran through the stacking gel, and 30 mA through the resolving gel. The end point was determined as when the loading dye ran off the gel.

Semi-dry transfer was completed using a PVDF membrane and a 3-buffer system containing cathode, anode I and anode II buffers. The transfer cell was run at 15 V for 35 minutes. After the transfer was complete, the membrane was briefly rinsed in distilled water before being stained with Ponceau S for 15 seconds to ascertain whether efficient transfer had occurred. The stain was then washed out with distilled water.

### Western blotting

The membrane was blocked in 10% skim milk in TBST overnight at 4°C. The following day, the membrane was washed in TBST. The membrane was then incubated in the primary polyclonal rabbit HSP60 (Sapphire Bioscience) 1: 250 in 5% skim milk in TBST overnight at 4° in a humidified chamber. After another set of washes in TBST, the membrane was incubated in peroxidase conjugated goat anti-rabbit IgG (Sigma) 1:1000 in 5% skim milk in TBST for 5 hours in a humidified chamber on a gyro rocker. After another set of washes in TBST on a gyro rocker, the membrane was transferred onto a glass plate and incubated for 1 minute with SuperSignal West Pico Chemiluminescent Substrate (Pierce). The membrane was visualised using the LAS-100 Plus Gel Documentation System (Fujifilm), and the intensity of the bands was measured using Gel Quant software. After stripping, the same blots were then probed with polyclonal rabbit HSP70 (Sapphire Bioscience). The Quant values generated for the proteins of interest were normalised to the house keeper gene actin after probing the same membrane with the anti-actin antibody.

### Statistical analysis

All statistical analysis in this study was carried out using Microsoft Excel. Data was averaged where appropriate, and the standard error of the mean (S.E.M.) was calculated using Excel. A two-tailed student’s t-test was carried out to determine the significance of the data, and the accepted level of significance was p < 0.05, which was denoted as ’*’.

## Results and discussion

### Cell culture and dose response curves

Cells treated with 50, 100 and 150 μM H_2_O_2_ and 1, 10 and 25 μM sodium azide showed growth rates comparable, if not greater than to that of untreated control cultures. Cells treated with 50 and 75 mM glucose showed a slightly reduced growth rate compared with the control. Finally the 100 and 125 mM glucose groups, the 200 and 250 μM H_2_O_2_, and the 50 and 100 μM sodium azide groups had considerably reduced growth rate through to the end of the culture period.

**Figure 1:**
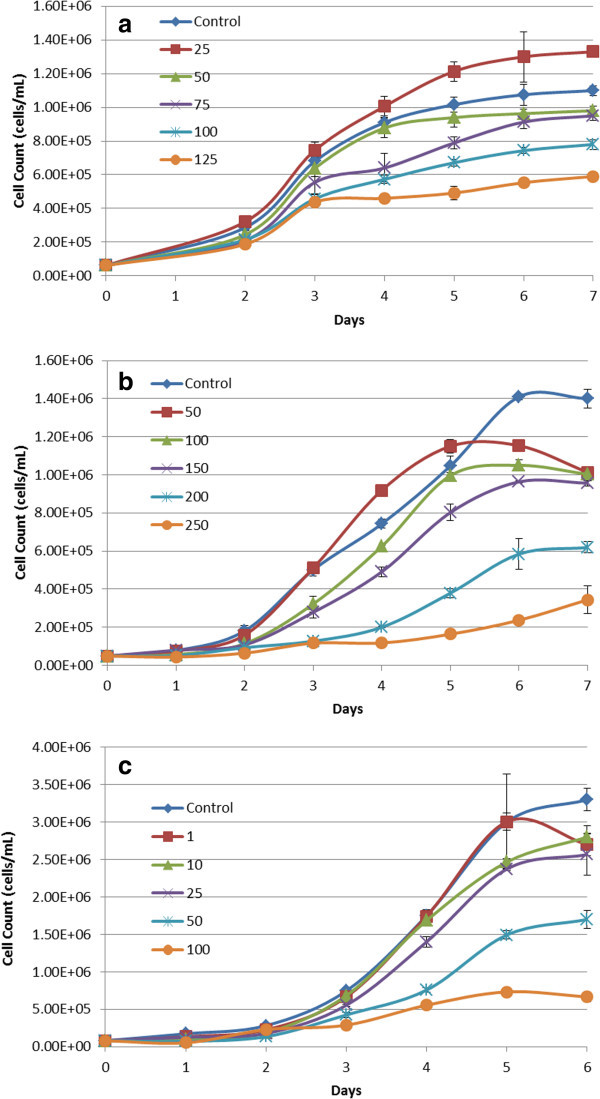
**Growth rates of HeLa cells supplemented with glucose, H2O2 and sodium azide. a**- Growth rate of HeLa cells supplemented with glucose (25-125 mM). Error bars showing S.E.M. The growth rate between days 3 and 5 has been taken as the growth rate during the exponential phase of growth. **b**- Growth rate of HeLa cells supplemented with H_2_O_2_ (0-250 μM). Error bars showing S.E.M. The growth rate between days 3 and 5 has been taken as the growth rate during the exponential phase of growth. **c**- Growth rate of HeLa cells supplemented with sodium azide (0-100 μM). Error bars showing S.E.M. The growth rate between days 3 and 5 has been taken as the growth rate during the exponential phase of growth.

To compare cell growth across treatments, the number of cells at each time point was normalised to the number of control cells. The rate of cell growth was determined during the exponential growth phase between days 3 and 5 of the culture period, and is exhibited in Figure 1a, b, and c.

As was to be expected, increasing the concentration of the treatment resulted in increased inhibition of cell growth. Slight growth inhibition resulted from treatments of 50 and 75 mM glucose (11% and 30% inhibition respectively), 150 μM H_2_O_2_ (4%), and 10 and 25 μM sodium azide (11% and 9% respectively). Strong growth inhibition resulted from treatments of 100 and 125 mM glucose (36% and 84% respectively), 200 and 250 μM H_2_O_2_ (54% and 91% respectively), and 50 and 100 μM sodium azide (47% and 78% respectively).

It is interesting that a degree of hormesis was present in all treatments. Hormesis is the name given to the stimulatory effects caused by low levels of potentially toxic agents (Calabrese et al., 2012). The 25 mM glucose, and 50 and 100 μM H_2_O_2_ treatments all resulted in increased growth rates compared to the control over the exponential growth phase. The 1 μM sodium azide was found to have a comparable growth rate to the control. While it may not be surprising that the comparatively low glucose levels result in increased growth, as it would convey a greater energy source to the cell culture, it is of interest that H_2_O_2_ does increase growth, which agrees with results of previous studies (Burdon, 1995). H_2_O_2_ and its stimulatory effect on cell proliferation have been of particular interest as it has been identified that the elevated levels of H_2_O_2_ that result from regular exercise have a hormetic effect, indicating that minor oxidative stress may have a beneficial effect (Radak et al., 2008).

To confirm that the impaired cell growth noted above was indeed a result of mitochondrial inhibition, and not necrotic cell death, the release of lactate dehydrogenase (LDH) into the growth media and formazan formation via the MTT assays as an indicator of mitochondrial dehydrogenase activity were carried out.

### LDH assay

**Figure 2:**
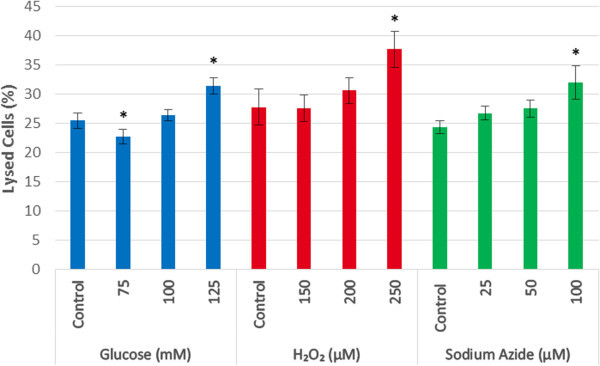
**Percentage of lysed cells in the presence of glucose, H**_**2**_**O**_**2**_**, and sodium azide.** The only concentrations that gave significantly elevated degrees of cell lysis compared to the control group were the maximum treatment concentrations. All other treatment concentrations had no significant difference in the degree of cell lysis. (* represents statistically significant values, p < 0.05).

After 3 days in culture, the percentage of LDH content relative to corresponding maximum control samples was calculated. It was assumed that every dead cell released an equal amount of LDH. Therefore, the percentage of LDH content was interpreted as the percentage of lysed cells, as shown in Figure 2.

The glucose control sample had 25.50 ± 1.31% lysed cells, and was not significantly different from the 75 and 100 mM glucose groups, which had 22.76 ± 1.29% and 26.46 ± 1.00% of lysed cells, respectively. However, treatment with 125 mM glucose resulted in a significantly increased (p < 0.05) percentage of lysed cells compared to the control; resulting in 31.46 ± 1.37% lysed cells.

The H_2_O_2_ control sample had 27.84 ± 3.04% lysed cells, and was not significantly different from the 150 and 200 μM H_2_O_2_ groups, which had 27.61 ± 2.33% and 30.69 ± 2.19% of lysed cells, respectively. However, samples treated with 250 μM H_2_O_2_ resulted in a significantly increased (p < 0.05) percentage of lysed cells compared to the control; resulting in 37.71 ± 3.09% lysed cells.

The sodium azide control sample had 24.42 ± 1.13% lysed cells, and was not significantly different from the 25 and 50 μM sodium azide groups, which had 26.80 ± 1.18% and 27.57 ± 1.51% of lysed cells, respectively. However, treatment with 100 μM sodium azide resulted in a significantly increased (p < 0.05) percentage of lysed cells compared to the control; resulting in 32.08 ± 2.85% lysed cells.

From the LDH assay results it can be concluded that 100 mM glucose, 200 μM hydrogen peroxide and 50 μM sodium azide do not result in significant cell lysis compared to control cells. These concentrations are of particular interest as they reduced growth rate but do not lead to an increase in cell lysis, indicative of a potential state of cellular stress. To determine if this stress is targeted at the level of the mitochondria MTT assays were implemented.

### MTT assay

**Figure 3:**
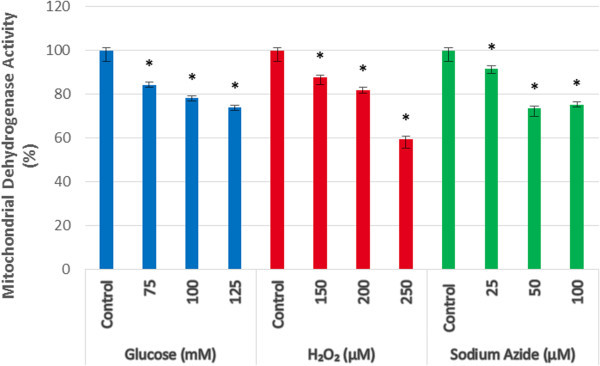
**Mitochondrial dehydrogenase activity in the presence of glucose, H**_**2**_**O**_**2**_**, and sodium azide.** All concentrations significantly inhibited mitochondrial bioenergetic function compared to respective control. (* represents statistically significant values, p < 0.05).

After 3 days in culture, cells were incubated with MTT for 2 hours. After which the formazan crystals were solubilised, the absorbance read, and results were interpreted as a percentage of mitochondrial activity relative to the control group, as seen in Figure 3.

All glucose samples were found to have significantly decreased (p < 0.05) mitochondrial dehydrogenase activity compared to control cells. 75 mM glucose resulted in 84.29 ± 1.20%, 100 mM resulted in 78.21 ± 1.13%, and 125 mM glucose resulted in 73.96 ± 1.12% mitochondrial dehydrogenase activity.

All H_2_O_2_ samples were found to have significantly decreased (p < 0.05) mitochondrial dehydrogenase activity relative to control cells. 150 μM H_2_O_2_ resulted in 87.70 ± 3.07%, 200 μM resulted in 81.97 ± 1.41%, and 250 μM resulted in 59.61 ± 4.21% mitochondrial dehydrogenase activity.

All sodium azide samples were found to have significantly decreased (p < 0.05) mitochondrial dehydrogenase activity relative to control cells. 25 μM sodium azide resulted in 91.76 ± 2.27%, 50 μM resulted in 73.61 ± 3.53%, and 100 μM resulted in 75.33 ± 1.20% mitochondrial dehydrogenase activity.

From the MTT assay results it can be concluded that 100 mM glucose, 200 μM hydrogen peroxide and 50 μM sodium azide do result in significant inhibition of mitochondrial bioenergetics compared to control cells. In conjunction with the dose response and LDH results, the MTT results indicate that 100 mM glucose, 200 μM hydrogen peroxide and 50 μM sodium azide result in mitochondrial specific cell stress. To further support this conclusion, DCFDA assays were used to investigate any ROS generation, a common association with mitochondrial impairment.

### DCFDA assay

**Figure 4:**
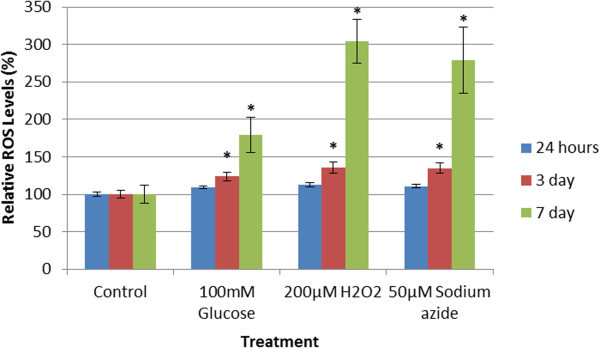
**ROS levels in the presence of glucose, H**_**2**_**O**_**2**_**, and sodium azide over 24 hours, 3, and 7 days.** Each sample contained significantly elevated ROS activity after 24 hours, which continued to rise over 3 and 7 days. A dramatic increase in ROS activity is observed between days 3 and 7. (* represents statistically significant values, p < 0.05).

After 1, 3, and 7 days in culture, cells were incubated with DCFDA for 1 hour. After which the fluorescence was read, and results were normalised to the H_2_O_2_ absent control. Results were interpreted as a percentage of ROS levels relative to the control group, as seen in Figure 4.

After 24 hours, all samples were found to have significantly increased (p < 0.05) ROS levels. The glucose treatment resulted in 109.06 ± 2.27%, the H_2_O_2_ treatment resulted in 112.93 ± 2.71%, and the sodium azide treatment resulted in 110.77 ± 2.24% ROS levels.

After 3 days, all samples were found to have significantly increased (p < 0.05) ROS levels. The glucose treatment resulted in 123.63 ± 6.24%, the H_2_O_2_ treatment resulted in 135.36 ± 7.33%, and the sodium azide treatment resulted in 134.77 ± 7.15% ROS levels.

After 7 days, all samples were found to have significantly increased (p < 0.05) ROS levels. The glucose treatment resulted in 179.95 ± 23.78%, the H_2_O_2_ treatment resulted in 304.56 ± 29.02%, and the sodium azide treatment resulted in 279.13 ± 43.82% ROS levels.

The DCFDA assay results show that increasing the time of exposure to each of the treatments increases the degree of intracellular ROS generation, supporting the conclusion that 100 mM glucose, 200 μM hydrogen peroxide, and 50 μM sodium azide result in mitochondrial specific cell stress. Increased levels of intracellular ROS activity have been shown to induce a concentration-dependent transactivation and DNA-binding activity of heat shock factor-1 (HSF-1), the principal transcription factor of HSP60 (Jacquier-Sarlin and Polla, 1996). Therefore it can be expected that if these treatments do induce a heat shock response, a longer treatment period will result in a more pronounced induction of HSP60. Western blots were implemented to investigate the effect of 100 mM glucose, 200 μM hydrogen peroxide, and 50 μM sodium azide on HSP60 & HSP70 expression.

### Western blotting

After total protein was separated on a polyacrylamide gel and transferred to a PVDF membrane, Ponceau S staining was used to show the standard HSP60 band to be at 60 kDa and that protein loading was even. After western blot, a single band for each sample was seen and the bands appeared to be the same size as the standard HSP60 protein band. The bands were then quantitated using the Gel Quant software, and the relative expression to the control was plotted.

**Figure 5:**
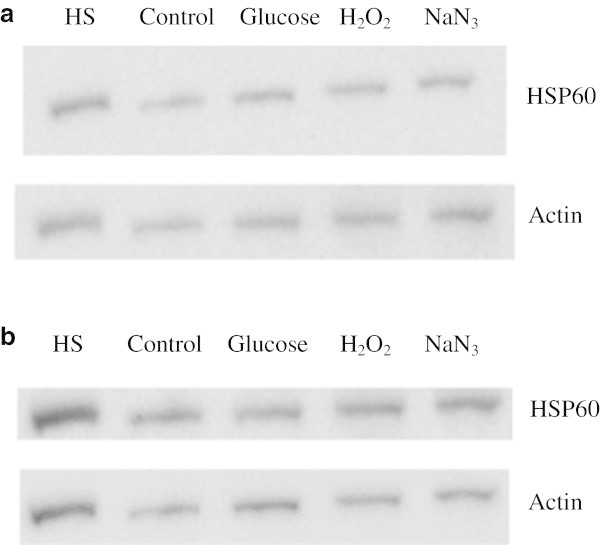
**Western blots of HSP60 expression in HeLa cells treated with heat shock, glucose, H2O2 and sodium azide over 3 and 7 days. a**- Western blots of HSP60 and actin in cells treated with heat shock, glucose (100 mM), H_2_O_2_ (200 μM), and sodium azide (50 μM), respectively, over 3 days. The greatest induction of HSP60 is observed in the sodium azide treated sample, with similar levels of HSP60 observed in the glucose and hydrogen peroxide treatments. HSP60 was tested for a minimum of three times on different blots to provide an accurate representation of its induction. **b**- Western blots of HSP60 and actin in cells treated with heat shock, glucose (100 mM), H_2_O_2_ (200 μM), and sodium azide (50 μM) over 7 days. The greatest induction of HSP60 is observed in the sodium azide treated sample, followed by the hydrogen peroxide treatment, with samples treated with glucose having a lesser degree of induction. HSP60 was tested for a minimum of three times on different blots to provide an accurate representation of its induction.

**Figure 6:**
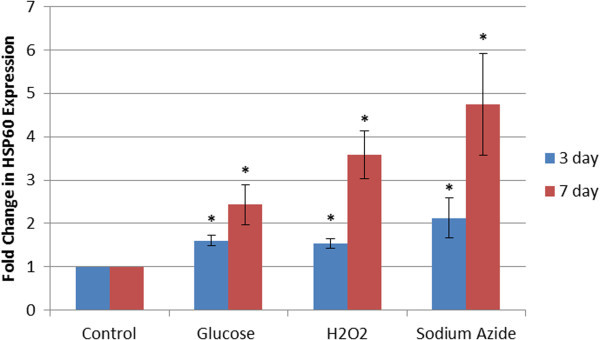
**Relative HSP60 expression in cells treated with heat shock, glucose (100 mM), H2O2 (200 μM), and sodium azide (50 μM) over both 3 and 7 days.** All treatments and time periods give a significantly increased level of expression of HSP60 compared to control samples. (* represents statistically significant values, p < 0.05). The fold change in HSP60 expression is even across all treatments after 3 days followed by a dramatic rise in expression levels after 7 days, a similar trend to that seen in the DCFDA assay.

HSP60 protein expression was found to be upregulated in all treatments over 3 days, as demonstrated in Figures 5a and 6. The heat shocked cells had a 2.23 fold increase in HSP60 expression, while the 100 mM glucose, 200 μM H_2_O_2_, and 50 μM sodium azide had 1.61, 1.54 and 2.12 fold increases respectively. All were significantly different to the control (p < 0.05).

Additionally, HSP60 protein expression was found to be further upregulated in all treatments over a 7 day treatment period, as shown in Figures 5b and 6. The heat shocked cells had a 2.38 fold increase in HSP60 expression according to these membranes. The 100 mM glucose, 200 μM H_2_O_2_, and 50 μM sodium azide had 2.43, 3.58 and 4.74 fold increases respectively, and each was significantly different from the control (p < 0.05).

The western blots for HSP60 show that treatment with glucose (100 mM), H_2_O_2_ (200 μM), and sodium azide (50 μM) all result in a significant increase in expression of HSP60. Length of exposure appears to have a pronounced effect on HSP60 induction, with the 7 day treatment having a marked increase in HSP60 expression. This drastic rise appears to mirror that seen in ROS activity over the same time period.

**Figure 7:**
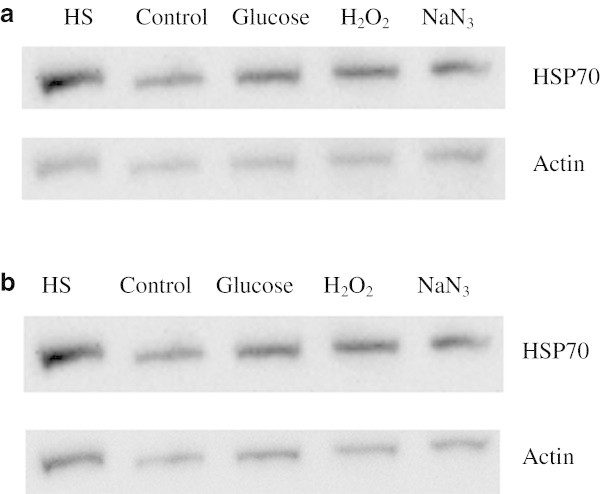
**Western blots of HSP70 expression in HeLa cells treated with heat shock, glucose, H2O2 and sodium azide over 3 and 7 days. a**- Western blots of HSP70 and actin in cells treated with heat shock, glucose (100 mM), H_2_O_2_ (200 μM), and sodium azide (50 μM) over 3 days. The greatest induction of HSP70 is observed in the sodium azide treated sample, followed by the hydrogen peroxide and glucose treated samples. HSP70 was tested for a minimum of three times on different blots to provide an accurate representation of its induction. **b**- Western blots of HSP70 and actin in cells treated with heat shock, glucose (100 mM), H_2_O_2_ (200 μM), and sodium azide (50 μM) over 7 days. The greatest induction of HSP70 is observed in the sodium azide treated sample, followed by the hydrogen peroxide treatment, with samples treated with glucose having a lesser degree of induction. HSP70 was tested for a minimum of three times on different blots to provide an accurate representation of its induction.

**Figure 8:**
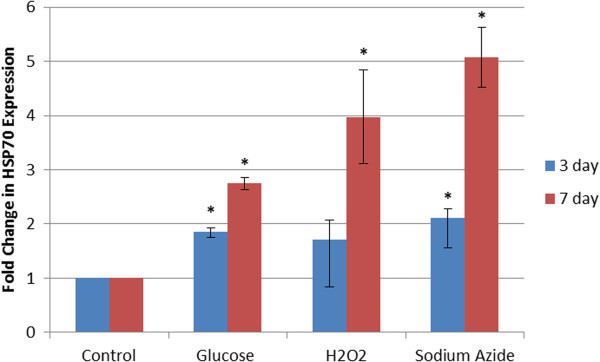
**Relative HSP70 expression in cells treated with heat shock, glucose (100 mM), H**_**2**_**O**_**2**_**(200 μM), and sodium azide (50 μM) over both 3 and 7 days.** All treatments and time periods give a significantly increased level of expression of HSP70 compared to control samples. (* represents statistically significant values, p < 0.05). The fold change in HSP70 expression is even across all treatments after 3 days followed by a dramatic rise in expression levels after 7 days, a similar trend to that seen in the DCFDA assay.

Even though the Gel Quant results for HSP60 induction are an average of three different blots, the band intensities seen in the images in Figure 5a and b are not entirely convincing. To further support the conclusion that the cellular stressors being investigated was indeed resulting in the induction of molecular stress proteins, the western blots were re-probed with antibodies against HSP70. Figures 7a,b, and 8 clearly provide additional evidence that a general cellular stress response is being evoked, resulting in a heat shock response from at least HSP60 and HSP70. The degree of induction of HSP70 closely mirrors that of HSP60.

HSP60 plays a critical role in the molecular cellular stress response targeted at the level of the mitochondrion. The primary role of HSPs is that of a molecular chaperone, where they act to mediate the folding, assembly or translocation across the intracellular membranes of other polypeptides; and a role in protein degradation, making up some of the essential components of the cytoplasmic ubiquitin-dependent degradative pathway (Burel et al., 1992). Additionally, when exposed to a various proteotoxic stressors, the expression of HSPs is induced in order to minimise cellular damage, as well as to stave off apoptosis, by stabilising compromised proteins (Santoro, 2000).

The inducible HSP component of a cell’s total HSP pool is regulated by HSFs, of which HSF-1 is the major regulator. In the absence of cellular stress, HSF-1 is inhibited due to its association with HSPs and is therefore maintained in an inactive state. If and when a cellular stress does occur, the HSPs bind to any misfolded proteins, and subsequently dissociate from HSF-1. This allows the HSF-1 monomers to oligomerise and form active trimers, regaining their DNA binding activity. The trimers undergo stress-induced serine phosphorylation and are translocated to the nucleus (Prahlad and Morimoto, 2008). Upon nuclear localisation, HSF-1 binds to the HSE situated upstream of heat shock responsive genes, which results in HSP gene transcription. However, the mechanisms for stress sensing and signalling to activate HSF-1 have not been fully elucidated. However, there is growing evidence in the literature that this mechanism is mediated by ROS, and in particular H_2_O_2_ (Ahn and Thiele, 2002).

H_2_O_2_ has been previously documented to induce a concentration-dependent transactivation and DNA-binding activity of HSF-1, although to a lesser extent than that of the classical heat shock treatment (Jacquier-Sarlin and Polla, 1996). Sensing of the oxidative stress requires two cysteine residues within the HSF-1 DNA-binding domain that are engaged in redox-sensitive disulfide bonds. HSF-1 derivatives in which either or both of these cysteine residues are mutated have been found to be defective in stress-inducible trimerization and DNA binding, stress-inducible nuclear translocation and HSP gene trans-activation, and in the protection of mouse cells from stress-induced apoptosis (Ahn and Thiele, 2002). In this way, H_2_O_2_ is thought to exerts two effects on the activation and the DNA-binding activity of HSF; H_2_O_2_ favours the nuclear translocation of HSF, while also altering the HSFs DNA-binding activity, which is achieved by oxidizing the two critical cysteine residues within the DNA-binding domain (Jacquier-Sarlin and Polla, 1996).

Hyperglycaemia has been reported to increase oxidative stress by increasing the rate of glycolysis while inhibiting oxidative phosphorylation (Crabtree, 1928). The build-up, and subsequent auto-oxidation, of glyceraldehyde-6-phosphate which ensues from increased glycolysis results in the generation of H_2_O_2_. As a result, the mitochondrion experiences an increase in ROS generation. As mentioned earlier, activation of HSF-1, the major regulator of HSPs, is redox dependent.

## Conclusion

This study demonstrated that hyperglycaemic conditions and oxidative stress can lead to the induction of HSP60 and HSP70expression, suggesting that the increased serum levels of these molecular stress proteins observed in T2DM patients could also be due to uncontrolled hyperglycaemia and oxidative stress. Interestingly there was also a significant and concomitant increase in the intracellular levels of ROS generated during the exposure of the HeLa cells to the stressors being investigated; suggesting that the induction of HSP70 & HSP60 was related to ROS mediated processes.
